# Retraction: Epithelial Membrane Protein-2 Promotes Endometrial Tumor Formation through Activation of FAK and Src

**DOI:** 10.1371/journal.pone.0276151

**Published:** 2022-10-11

**Authors:** The PLOS ONE Editors

After this article [1] was published, concerns were raised about western blot images in Figures 3, 4, and 6. Specifically:

- In Figure 3A in this article [1]:
  - The EMP2 panel appears similar to the EMP2 panel in Figure 2 in article [2] when vertically stretched and to the EMP2 panel in Figure 4B in article [3].
  - The β-actin panel appears similar to the β-actin panel in Figure 2 in article [2].
- In Figure 3B, the FAK panel appears to show a vertical discontinuity.
- In Figure 4B, there appear to be vertical discontinuities between lanes 1 and 2 in all panels and between lanes 2 and 3 in the P-FAK panel.
- In the EMP2 panel in Figure 6A, there appear to be similarities between regions in the background of lanes 5 and 6.
- In Figure 6C in this article [1]:
  - The total FAK panel for HEC-1A/EMP2 and for HEC-1A/V appear similar when flipped vertically.
  - The EEA1 panel for HEC-1A/V and for HEC-1A/RIBO have vertical discontinuities between lanes 5 and 6.

In editorial follow up on these issues, underlying images from replicate experiments were provided for Figures 3A and 3B. The corresponding author stated that the results look similar to those reported in [3] as they are using the same cell lines and antibodies, and that they believe the vertical discontinuity is likely an artefact of the development used with saran wrap covering the blots. The corresponding author additionally stated that the original underlying data for Figure 4B could not be located. The *PLOS ONE* Editors consider that the Figure 3A and 4B concerns cannot be resolved without the underlying data from the original experiments.

An underlying image from the original experiments was provided for the EMP2 panel in Figure 6A. This consisted of one blot for lanes 1–5, and one blot for lanes 6–8 which had been joined to create the EMP2 panel in Figure 6A, rather than being shown as separate panels. Therefore, whilst support is provided for the results, the *PLOS ONE* Editors have remaining concerns about the nature of the image issues.

Underlying images from the original experiments were provided for Figure 6C, with the exception of the EMP2 panel for HEC-1A/RIBO for which underlying images from replicate experiments were provided. However, the *PLOS ONE* Editors consider that the underlying image provided for the Total FAK panel for HEC-1A/EMP2 does not appear to match the blot shown in the published image. The above concerns about the total FAK panels for HEC-1A/EMP2 and HEC-1A/V in Figure 6C are therefore not fully resolved. The corresponding author stated that the blots for total FAK are similar in the two cell lines. The *PLOS ONE* Editors also consider that lanes 5 and 6 in the underlying image provided for the EEA1 panel for HEC-1A/V do not appear to match lanes 5 and 6 in the published image. The above concerns about the EEA1 panel for HEC-1A/V in Figure 6C are therefore not fully resolved. The underlying image was not supportive of the above concern about the EEA1 panel for HEC-1A/RIBO.

In light of the above issues, some of which could not be resolved in the absence of much of the original underlying data for Figures 3A, 3B, 4B, 6A, and 6C, the *PLOS ONE* Editors retract this article.

MF, RR, DS, CPH, ZR, SM, LKG, JB, and LG either could not be reached or did not respond. MW initially responded to the concerns, but did not respond to the final retraction decision.

Owing to the concerns about similarities with previously published content [2], published 2005 Elsevier which is not offered under a CC-BY license, the EMP2 and β-actin panels of Figure 3A are excluded from this article’s [1] license. At the time of retraction, the article [1] was republished to note this exclusion in the Figure 3A legend and the article’s copyright statement.
